# Acute lymphoblastic leukemia presenting with limping in a young adult

**DOI:** 10.1002/ccr3.5426

**Published:** 2022-02-23

**Authors:** Mhd Baraa Habib, Bashar Tannos, Mohamed Abdelrazek, Mohamed A. Yassin

**Affiliations:** ^1^ 36977 Internal Medicine Department Hamad Medical Corporation Doha Qatar; ^2^ 36977 Radiology Department Hamad Medical Corporation Doha Qatar; ^3^ 36977 Hematology Department Hamad Medical Corporation Doha Qatar

**Keywords:** acute lymphoblastic leukemia, back pain, initial presentation, limping, osteolytic lesions

## Abstract

Acute lymphoblastic leukemia is one of the rare malignancies in adult. We report a 29‐year‐old man presented with progressive limping followed a chronic back pain. Imaging showed reduced vertebral body height and diffuse lytic skeletal infiltration. Bone marrow aspiration confirmed B‐acute lymphoblastic leukemia/lymphoma, and the patient was treated with chemotherapy.

## Graphical abstract

ALL is a rare disease in adults and can present with symptoms or complications of diffuse lytic bone lesions. Physicians should have high index of suspicion to figure out such unusual challenging presentation, so the treatment will not be delayed.

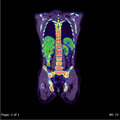

## 1 INTRODUCTION

Acute lymphoblastic leukemia/lymphoma (ALL) is primarily a disease of young children <6 years old. Most adult patients are over the age of 65. It is a rare disease with annual incidence worldwide of 1–5 cases/100,000 population.^1^ Most patients have findings associated with anemia, neutropenia, and/or thrombocytopenia due to bone marrow involvement. Symptoms may include infections, fatigue, or bleeding. Bone pain or arthralgias, fever, weight loss, and night sweats are also common in adults. B‐acute lymphoblastic leukemia/lymphoma (B‐ALL) may be suspected in a patient with circulating lymphoblasts or unexplained cytopenias.^1^ The diagnosis is usually confirmed by immunophenotype of bone marrow aspiration.

Lytic bone lesion is a result of many primary or secondary bone diseases which can be benign or malignant. Common causes of malignant osteolytic lesions are multiple myeloma, lymphoma, breast or lung cancer metastases, and rarely leukemia. Although there are no clear data in adults, the prevalence of osteolytic lesions is low when was investigated in pediatric population.^2^, ^3^

Here, we are reporting a case of a patient who came with lower limbs weakness and chronic progressive back pain. Images showed diffuse osteolytic lesions; then finally, he ended with diagnosis of B‐ALL.

## 2 CASE REPORT

A 29‐year‐old male patient, not known to have any chronic illness, presented to emergency department complaining of difficulty in walking over the past few days. It was proceeded by five‐month history of back pain. He has had recurrent visits to ED where he received symptomatic treatment. However, his pain got worse last month. He also mentioned mild unmeasured weight loss. He denied any history of trauma or falling down, and there was no change in his bowel or urine habits.

Physical examination revealed mild bilateral proximal lower limbs weakness and restriction of passive movements because of back pain. Knee tendon reflexes were brisk, with down going planters. There was tenderness in the lower thoracic and lumbar spine. Otherwise, the examination has not revealed any abnormal findings.

**FIGURE 1:**
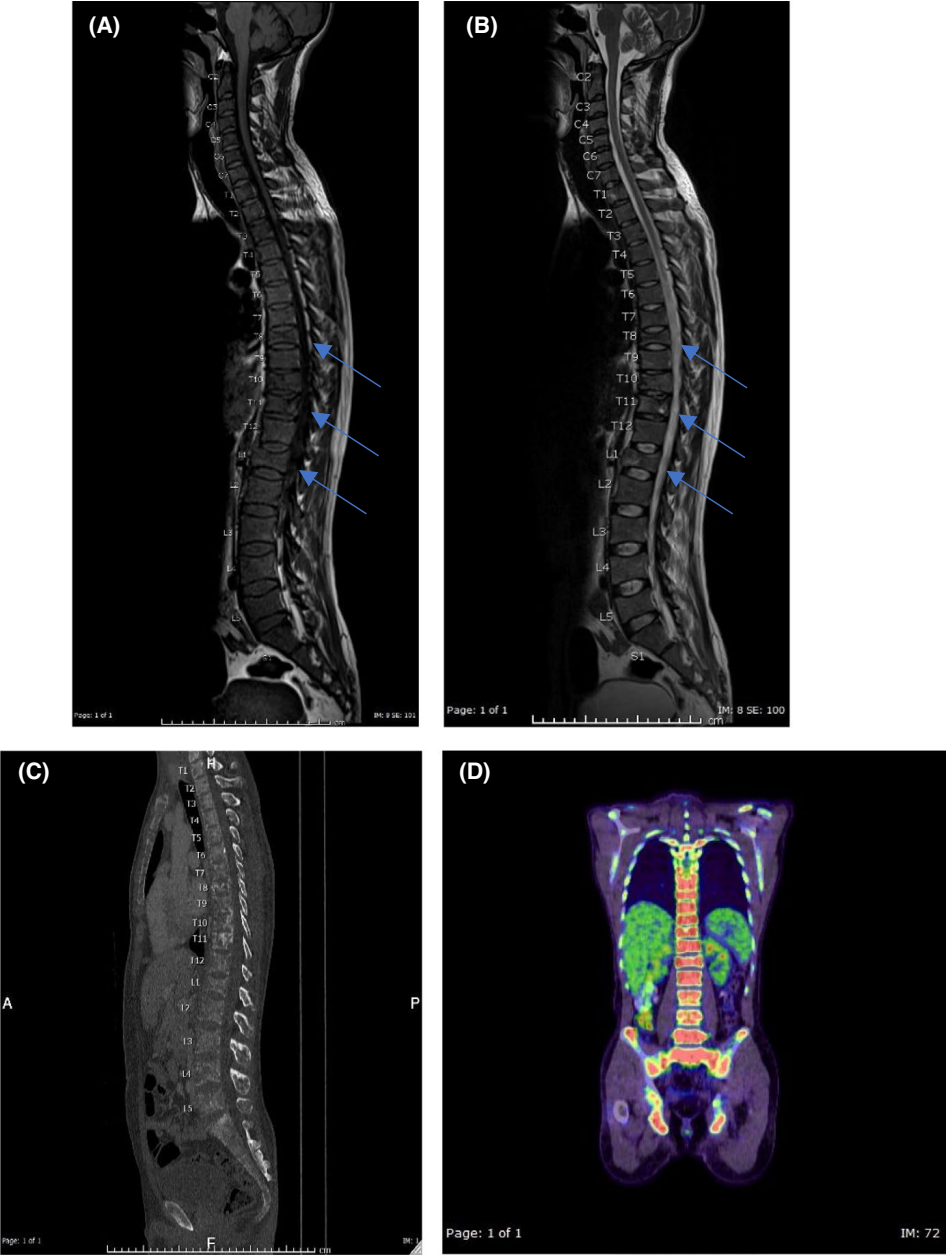
(A) Sagittal T1‐WI of the whole spine and B) Sagittal T2‐ WI of whole spine show heterogenous appearance of bone marrow of the spine with relative diffuse low signal intensity of bone marrow in T1 ‐WI and reduced height of multiple vertebral bodies affecting mainly T8, T11 and L1 (Arrows in A &B). (C) Sagittal reformatted Computed Tomography image of the Thoracic and lumbosacral spine in bone window setting show diffuse mainly lytic bony lesions involving the scanned spine and sternum with redemostration of reduced height of T8, T11 and L1. (D) Coronal color‐coded PET‐Scan image of the whole body show increased tracer uptake in the whole spine and the visualized parts of the pelvic bones (red color in D)

Thoracolumbar spine X‐ray showed diffuse decreased bone density with decrease lower thoracic and lumbar spine vertebral body height of D8 and L1. MRI spine showed no evidence of cord compression or mass lesion, but heterogenous thoracic bone marrow signal intensity with relative T1WI hypointensity and multilevel reduced vertebral body height (see images A and B). CT chest and abdomen with contrast showed diffuse lytic skeletal infiltration involving most of the axial skeletal bones included in the scan including sternum, ribs, vertebral column, and pelvic bones consistent with systemic bone infiltrating disease (see image C). FDG PET scan showed extensive lytic lesions throughout the skeleton with focal increased tracer uptake manifest an extensive malignant process (see image D) (Figure 1).

Laboratory tests showed anemia with low reticulocytes count, hypercalcemia, low PTH, and high CRP. Iron panel and multiple myeloma work‐up were negative (see Table 1). Peripheral smear showed moderate normocytic hypochromia anemia with scattered tear drops and rare nucleated red blood cells, as well as some reactive lymphocytes with few atypical forms encountered, occasional pelgeroid neutrophils and myelocytes spotted, and rare blast spotted. Flow cytometry analysis on bone marrow aspirate showed approximately 36% blast cells with immunophenotype profile consistent with precursor B‐ALL. The diagnosis of ALL was confirmed, and the patient was started on steroids, BFM protocol which contains prednisolone, vincristine, daunorubicin, and L‐asparaginase. Morphine was used as a painkiller.

**TABLE 1:** Laboratory test results upon admission

Detail	Value w/units	Normal range
Beta 2 microglobulin	2.72 mg/L	0.80–2.20
Retic %	1.3%	0.5–2.5
LDH	248 U/L	135–225
Fe% saturation	28%	15–45
WBC	7.5 × 10^3^/μl	4.0–10.0
Hgb	10.0 gm/dl	13.0–17.0
MCV	81.6 fL	83.0–101.0
Platelet	157 × 10^3^/μL	150–400
Urea	8.8 mmol/L	3.2–7.4
Creatinine	89 μmol/L	64–110
Sodium	138 mmol/L	135–145
Potassium	4.6 mmol/L	3.6–5.1
Adjusted calcium	2.67 mmol/L	2.10–2.55
Alk Phos	77 U/L	40–150
ALT	8 U/L	0–55
CRP	89 mg/L	0–5
Ferritin	489.0 μg/L	48.0–420.0
Vit D	19 ng/ml	<20 ng/ml to deficiency
PTH	9 pg/ml	15–65

## 3 DISCUSSION

Acute lymphoblastic leukemia/lymphoma is a rare disease in adult population, and most studies were conducted in pediatrics. It can present in many scenarios. However, patients often complain of constitutional symptoms such as fever and weight loss, lymphadenopathy, or even bone pain.^1^, ^4^ Musculoskeletal manifestations are frequent in patients with ALL. Moreover, bone pain was the presenting symptom of 40 percent of children having ALL as reported in one study.^5^ Hypercalcemia and the abnormal bone metabolic effects have been contributed to several factors: paraneoplastic production of humoral factors especially PTHrP, direct bone destruction by the malignant cells, and the local cytokines effect. However, there is no clear definite reason so far and it looks multifactorial.^6^ Reported skeletal radiographic findings in children with ALL include metaphyseal banding, periosteal reactions, osteolysis, osteosclerosis, and osteopenia.^7^ Although the combination of limp, chronic back pain, and osteolytic bone lesions can be the presenting manifestation of myeloma and some solid cancer metastases,^8^ it is unusual in ALL.

In our patient, the findings of back pain, hypercalcemia, and lytic bone lesions draw the physician attention toward multiple myeloma and solid cancer lytic metastases. Therefore, serum and urine electrophoresis were sent and came back negative. Ct scan of chest, abdomen, and pelvis was done looking for possible metastatic primary solid cancer; however, it did not show any mass lesion, but support the finding of MRI of diffuse osteolytic lesions and compressing fractures. Finally, patient underwent bone marrow aspiration and flow cytometry which showed approximately 36% blast cells with immunophenotype profile consistent with precursor B‐acute lymphoblastic leukemia (B‐ALL). This challenging presentation may mislead the diagnosis and sometimes delay the treatment.

## 4 CONCLUSION

Since ALL is a very rare disease in middle age adults, and diffuse lytic bone lesions are not a quiet common manifestation of this disease, it may result in a challenge to the physician to reach the right diagnosis. Hence, we advise physician facing such cases to have high index of suspicion to figure out this unusual presentation. Therefore, earlier treatment can be started and give the patient more change of improving.

## CONFLICT OF INTEREST

The authors report no conflict of interest.

## AUTHOR CONTRIBUTIONS

The first (MBH) and other authors (BT), (MA), and (MY) all contributed to the writing and preparation of this article. MBH written the initial draft of the manuscript and attempted the literature review. The draft was revised and updated by MY. MBH and BT were part of the medical treating team and did the follow up. MA added the appropriate radiological images to the case. All the authors critically reviewed the initial and the final draft of the manuscript and approved it for submission.

## ETHICAL APPROVAL

The publication of this case report was approved by local medical research committee/institutional review board.

## CONSENT

Written informed consent was obtained from the patient to publish this case.

## Data Availability

The data that support the findings of this study are available from author MBH upon a reasonable request.
